# Vacuum-assisted excision: one-step approach to the diagnosis and percutaneous treatment of small early breast cancer (the VAE-BREAST 01 study protocol)

**DOI:** 10.3389/fonc.2025.1687634

**Published:** 2025-10-24

**Authors:** Henrique Lima Couto, Bertha Andrade Coelho, Bernardo Ferreira de Paula Ricardo, Paola Hartung Toppa, Aleida Nazareth Soares, Bruna Torres Silvestre da Silva, Douglas de Miranda Pires, Tereza Cristina de Oliveira Ferreira, Paula Clarke, Shirley das Graças Ferreira, Larissa Barbosa Oliveira, Romana Giordani Ribeiro Saliba, Paula Cristina Martins Soares, Thais Paiva Moraes, Ana Carolina Guglielmelli Mendonça, Amanda Cristina Braga de Oliveira, Daniela Rodrigues Siqueira, Jane Sanglard de Oliveira, Charles Andreé Joseph de Padua, Geraldo Felício Cunha, Marcus Simões Castilho, Bárbara Pace Silva Assis Carvalho, Gabriel de Almeida Silva, Waldeir José de Almeida Júnior, Clecio Ênio Murta de Lucena, Eduardo Carvalho Pessoa, Annamaria Massahud Rodrigues dos Santos, Heverton Leal Ernesto de Amorim, Ruffo Freitas-Junior, Marcus Nascimento Borges, Andre Mattar, Marcelo Antonini, Daniel de Araújo Brito Buttros, Lorena Lima Coto Dominguez, Bruna Pires, Carolina Nazareth Valadares, Fernando Marcos dos Reis

**Affiliations:** ^1^ Redimama-Redimasto, Belo Horizonte, Brazil; ^2^ Brazilian Society of Mastology, Belo Horizonte, Brazil; ^3^ UNIFIPMOC University Center, Montes Claros, Brazil; ^4^ MaterMOC Breast Unit, Montes Claros, Brazil; ^5^ Anatomia Laboratory, Belo Horizonte, Brazil; ^6^ Faculty of Medical Sciences of Minas Gerais, Belo Horizonte, Brazil; ^7^ Analysis Laboratory, Belo Horizonte, Brazil; ^8^ Santa Casa College BH, Belo Horizonte, Brazil; ^9^ Santa Casa de Misericordia de Belo Horizonte, Belo Horizonte, Brazil; ^10^ Military Hospital (ISPM), Belo Horizonte, Brazil; ^11^ Mater Dei Hospital, Belo Horizonte, Brazil; ^12^ Cetus Oncology Belo Horizonte, Belo Horizonte, Brazil; ^13^ Radiocare, Belo Horizonte, Brazil; ^14^ Oncoclinicas, Belo Horizonte, Brazil; ^15^ Diagnostic Imaging Sonar, Belo Horizonte, Brazil; ^16^ Orizonti Institute, Belo Horizonte, Brazil; ^17^ Federal University of Minas Gerais, Belo Horizonte, Brazil; ^18^ São Paulo State University Júlio de Mesquita Filho, School of Medicine, Botucatu, Brazil; ^19^ Minas Gerais State Servants Welfare Institute, Belo Horizonte, Brazil; ^20^ UD Diagnosis, João Pessoa, Brazil; ^21^ Federal University of Goiás, Goiânia, Brazil; ^22^ Women Hospital, São Paulo, Brazil; ^23^ Hospital of the State Public Servant of São Paulo, São Paulo, Brazil; ^24^ Claretian University Center, Rio Claro, Brazil; ^25^ Hospital Paulistano, São Paulo, Brazil

**Keywords:** vacuum assisted excision, minimally invasive treatment, early breast cancer, de-escalation, precision oncology

## Abstract

**Introduction:**

Vacuum-assisted excision (VAE) of breast lesions is a technique used for diagnostic and therapeutic purposes and is performed on an outpatient basis, with local anesthesia and image guidance. Currently, VAE is used in the management of benign lesions and lesions of uncertain malignant potential (B3 lesions). More recently, there has been interest in VAE for the percutaneous treatment of small breast cancers, the aim of which was to reduce morbidity and aggressive surgical treatment. Due to how conventional VAE is performed, histopathological assessment of the resection margins is not possible. Obtaining free margins after a breast cancer resection is a primary objective in the surgical treatment of this disease. If VAE could ensure free margins and the absence of residual tumor in the surgical excision, it would represent a safe method for a minimally invasive treatment, providing an effective percutaneous treatment of small early breast cancers.

**Methods:**

The prospective VAE-BREAST 01 study explores the role of VAE associated with cavity margin sample shaving (CMSH) as a one-step approach in the diagnosis and complete excision of small breast tumors, ensuring the absence of residual disease in surgical pathology. Women with lesions smaller than 1.5 cm, ACR BI-RADS™ (American College of Radiology Breast Imaging Reporting and Data System) category 4 or 5, and identified by screening or clinical alteration are included. Multifocal, multicentric breast cancers and breast cancers associated with diffuse and extensive calcifications are excluded. The sensitivity, specificity, accuracy, positive predictive value, negative predictive value, and the false-negative and false-positive rates of VAE+CMSH for the complete excision of breast cancers will be calculated. The collected data also will include patients’ demographics, image characteristics of the lesions, information regarding the VAE+CMSH and surgical procedure, biopsy and surgical pathology, and data on side effects, patient acceptance, cosmetic results, and patients’ experiences during VAE.

**Ethics and dissemination:**

Ethics approval was obtained from the Brazilian National Research Ethics Commission (CONEP). Participants will provide written informed consent, and researchers will follow institutional guidelines for data collection and management.

**Clinical Trial Registration:**

https://ensaiosclinicos.gov.br/, identifier U1111-1301-4235

## Highlights

- Our prospective study will provide valuable information on the potential role of VAE+CMSH in the percutaneous treatment of small breast cancers.

## Introduction

Breast cancer is an extremely heterogeneous and multifactorial disease. With the establishment of systematic population screening, the majority of diagnosed tumors by screening are small and non-palpable. Screening mammography has been associated with a moderate reduction in mortality from breast cancer in women aged 40–70 years (1–3). Benchmarks reported by the Breast Cancer Surveillance Consortium for mammography screening include a median tumor size of 14 mm, 77.3% node-negative cancers, 52.6% minimal cancers (<1 cm invasive cancers or *in situ*), and 74.8% stage 0 and 1 cancers (4).

Breast cancer treatment has undergone numerous changes and advances, leading to the current era of personalized and precision treatment (5). The establishment of mammographic screening programs has increased the diagnosis of small breast cancers, many of which have favorable biological characteristics. Some of these tumors have excellent long-term outcomes, with the 10-year breast cancer-specific survival approaching 100% (1). Such tumors may never become symptomatic within a patient’s lifetime due to their indolent nature may thus represent overdiagnosis (3, 6, 7). It is estimated that, for every breast cancer death prevented by screening, three cancers were overdiagnosed and consequently overtreated (3, 4, 6, 7). There still remains no method to identify which cancers are likely to be overtreated. Thus, there is increasing interest in the de-escalation of locoregional therapies for small screen-detected breast cancers.

It is crucial to find a balance between early detection and the treatments offered, promoting a more personalized and balanced approach to the management of breast lesions. Recently, the omission of sentinel node biopsy (SNB) has been recognized as a standard approach for early T1N0(us) breast cancers with good prognosis in women over 50 years, with important reduction of the harms of surgical treatment (8–13). It is of utmost importance to note that the majority of cancers detected in screening programs are small, node-negative, and hormone receptor-positive cancers eligible for SNB omission (4, 11).

Diagnostic imaging and percutaneous interventions are increasingly playing a key role in the management of patients with breast abnormalities, from fibroadenoma to lesions of uncertain malignant potential (B3 lesions) in core needle biopsy (14–20). Vacuum-assisted excision (VAE) can completely excise small breast cancers. However, until now, it is not possible to ensure complete excision with free margins without conventional surgery (21).

SMALL is a prospective, multicenter, randomized phase III trial of VAE *versus* surgery in patients with small, biologically favorable, screen-detected invasive breast cancer. VAE could potentially reduce the morbidity and surgical overtreatment of screen-detected estrogen cancers with good prognosis (22). However, with conventional VAE, it is currently not possible to assess the margin status. This new trial aimed to evaluate the efficiency of VAE combined with percutaneous cavity margin sample shaving (CMSH) in predicting complete excision of breast cancers smaller than 1.5 cm using a one-step diagnostic–treatment approach for ACR BI-RADS™ (American College of Radiology Breast Imaging Reporting and Data System) category 4 or 5 lesions (23).

## Methods

### Study design

**Figure 1:**
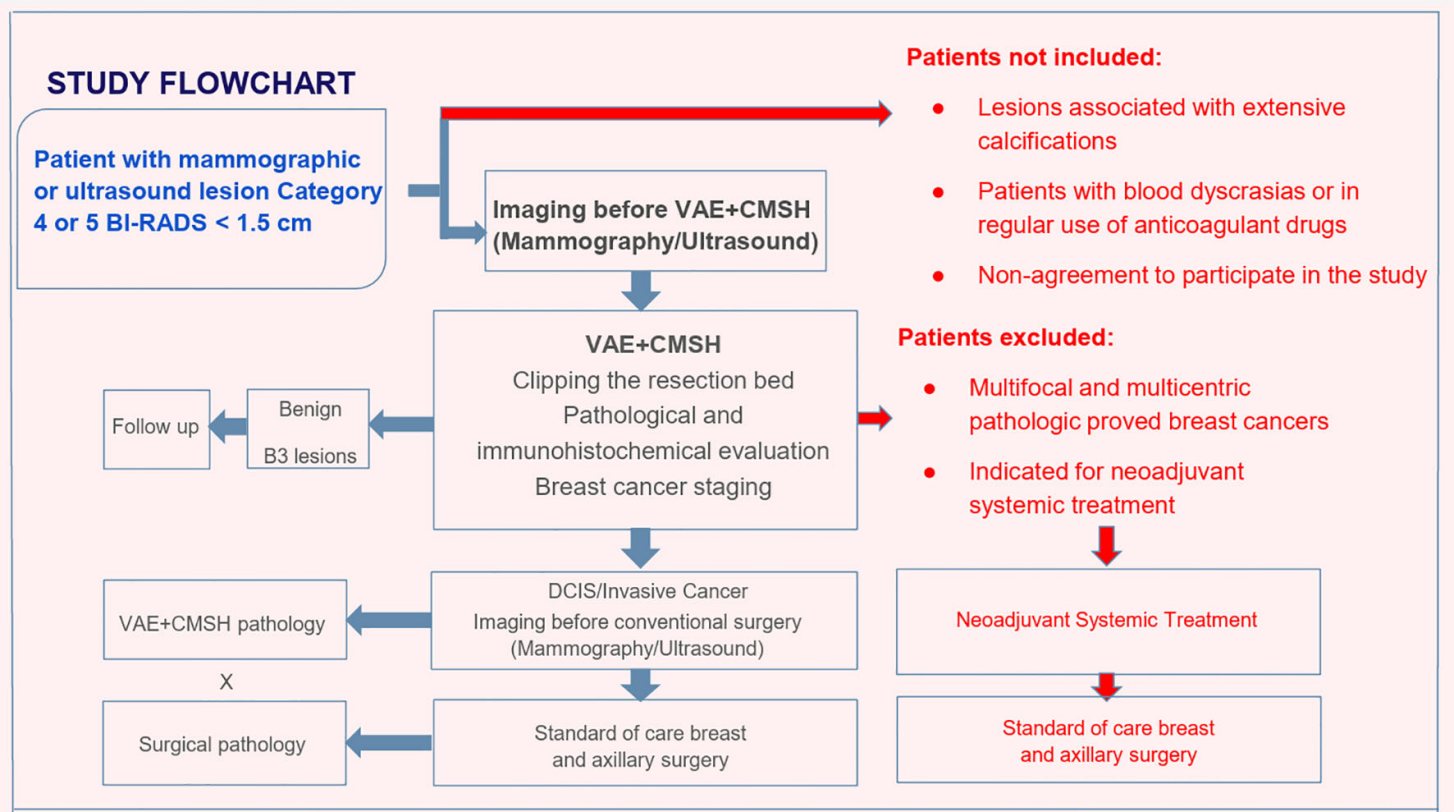
Study flowchart. *VAE*, vacuum-assisted excision; *CMSH*, cavity margin sample shaving; *B3 lesions*, lesions of uncertain malignant potential in core needle biopsy; *DCIS*, ductal carcinoma *in situ*.

This is a phase 2, prospective, non-randomized clinical trial recruiting patients with ACR BI-RADS™ category 4 or 5 mammographic or sonographic breast lesions smaller than 1.5 cm (**Figure 1**). The aim was to evaluate the efficiency of VAE+CMSH in predicting complete excision of breast cancers [invasive cancers (IC) and ductal carcinoma *in situ* (DCIS)] smaller than 1.5 cm in a one-step diagnostic–treatment approach for ACR BI-RADS™ category 4 or 5 lesions. The CMSH immediately after VAE is a diagnostic test to predict complete percutaneous excision and will be evaluated based on the sensitivity, specificity, accuracy, positive predictive value (PPV), negative predictive value (NPV), false-negative rate (FNR), and false-positive rate (FPR). The CMSH will be considered the diagnostic test in the evaluation and the surgery the gold standard. CMSH negative (no tumor cells in the samples) or positive (with tumor cells in the samples) results will be compared with the gold standard excision/surgery negative (no residual tumor cells) or positive (presence of residual tumor cells) results.

### Study setting

The procedures are realized in an outpatient basis at two Breast Units in the state of Minas Gerais, Brazil.

### Study duration

The first patient was recruited on November 29, 2023. Recruitment is estimated to end in December 2026.

### Eligibility criteria

Women with ACR BI-RADS™ category 4 or 5 lesions smaller than 1.5 cm identified by screening or clinical alteration, who are literate, and are aged over 18 years are included. The exclusion criteria were: multifocal and multicentric pathologically proven breast cancers, lesions associated with diffuse and extensive calcifications, patients with blood dyscrasias or in regular use of anticoagulant drugs, and non-agreement to participation in the study. Patients indicated for neoadjuvant systemic treatment after VAE+CMSH are also excluded (**Figure 1**).

### Interventions and patient pathways

Patients with mammographic or sonographic lesions (≤1.5 cm) classified as ACR BI-RADS categories 4–5 will undergo VAE+CMSH (**Figure 1**).

If the pathological diagnosis reveals a benign lesion, the patient is discharged and returned to routine screening. If the pathological diagnosis indicates a B3 lesion, the case is discussed by a multidisciplinary team, and the patient is preferably monitored or undergoes surgical excision, in case of imaging–pathology discordance. All types of B3 lesions are allowed to be followed without surgical excision. The final multidisciplinary decision is absolute.

If malignancy is diagnosed (DCIS or IC), the patient is staged and submitted to standard primary surgical treatment, regardless of the CMSH result, according to the Brazilian Guideline for Breast Cancer Diagnosis and Treatment from the Brazilian Health Department (24), and the surgical pathology is compared with the VAE+CMSH pathology.

Patients with pathologically confirmed multifocal or multicentric disease in the preoperative stage are excluded. Patients indicated for neoadjuvant systemic treatment, according to the Brazilian Guideline for Breast Cancer Diagnosis and Treatment from the Brazilian Health Department (24), after VAE+CMSH are also excluded.

### Imaging

All patients are submitted to mammography and breast ultrasound before VAE+CMSH. Only those with ≤1.5-cm lesions classified as ACR BI-RADS categories 4–5 in both methods will undergo VAE+CMSH. If two lesions are detected in a patient, these are recorded as individual lesions, unless they proven to be multifocal or multicentric breast cancer, in which case they are excluded (**Figure 1**). Magnetic resonance imaging (MRI) of the breast or contrast-enhanced mammography (CEM) is not considered in the inclusion or exclusion criteria and is allowed at the discretion of the multidisciplinary team in charge. If requested at any time, the lesion measurement in the MRI or CEM is not applied as an inclusion or an exclusion criterion.

### VAE+CMSH standard procedure

All procedures are performed by mastologists or breast-dedicated radiologists experienced in VAE by ultrasound or stereotactically. In Brazil, mastology is a specialty. Brazilian mastologists are trained and qualified physicians in the specialty of mastology with the skills to study, prevent, diagnose, and treat diseases, congenital and/or acquired conditions of the breasts, promoting and executing the necessary therapeutic means, whether clinical, surgical, reparative, and/or palliative. Mastologists master the execution of fine needle aspiration (FNA) biopsy, core needle biopsy (CNB), vacuum-assisted biopsy (VAB), and/or VAE, guided or unguided by imaging methods (25).

**Figure 2:**
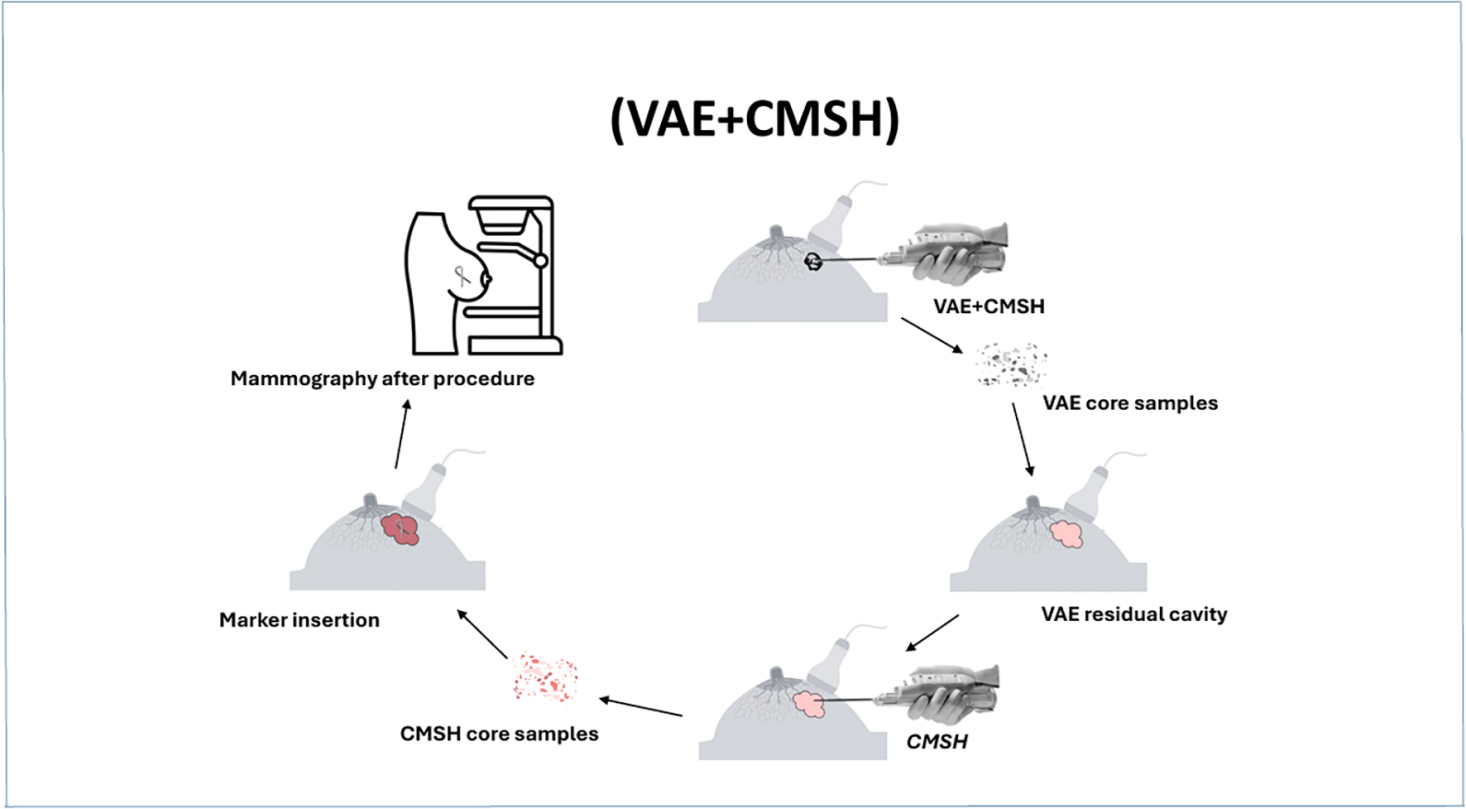
Standard protocol of vacuum-assisted excision (VAE) and cavity margin sample shaving (CMSH).

The VAE+CMSH procedure is always performed on an outpatient basis with local anesthesia. A 7-G or a 10-G needle is used at the discretion of the performing physician according to each case and patient features, such as distance from the skin and the pectoralis muscle and implants, among others (**Figure 2**).

The VAE step of the procedure consists in carrying out the number of core samples necessary for the complete excision of the lesion. The excision is always performed in round circles of 12 samples, and so on, with the Encore Inspire™ device. The VAE step ends with the last round circle after removal of the lesion. The number of VAE samples is recorded in the report and then sent to the pathologist in a special bottle labeled VAE.

**Figure 3:**
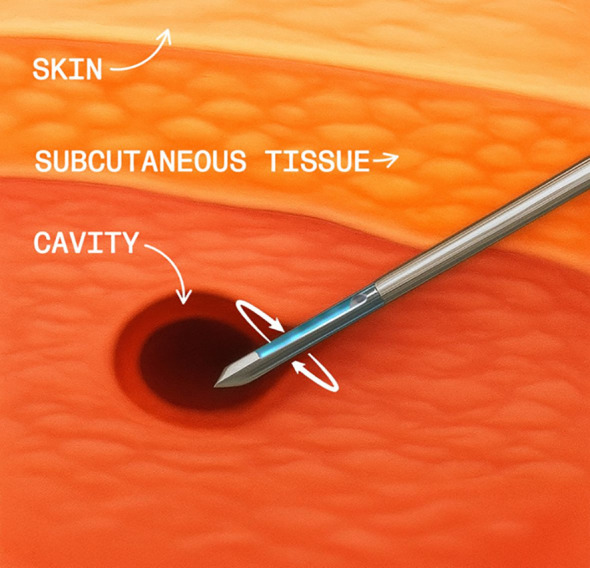
Cavity margin sample shaving (CMSH).

CMSH consists of, after documenting the complete excision of the lesion, rinsing the needle (when guided by ultrasound), performing another round of 12 core samples, and sending these to the pathologist in a separate bottle as CMSH evaluation (**Figure 3**). Evidently, when performed stereotactically, the step of rinsing the needle is not required. The biopsy site is then clipped, followed by immediate mammographic confirmation of clip placement and recording any complications and the procedure time.

### Surgical procedure

Surgical excision is mandatory and is performed for all cases of malignancy (DCIS/IC) in VAE+CMSH. After surgery, the surgical specimen is radiographed to confirm the presence of the marker placed during VAE+CMSH. Surgical treatment, breast conservation or mastectomy, and axillary surgery are performed according to clinical practice and standard of surgical care. Patients are submitted to no axillary surgery (omission of SNB), SNB, or axillary clearance according to standard of care (11, 24).

### VAE+CMSH pathology

Gross specimens are separated from the clots, measured, weighed, and inked. Total inclusion of the fragments is performed, and slices are cut every 4 μm. Tests range from the usual hematoxylin–eosin (HE) analysis on slides, with or without immunohistochemistry and at the discretion of the case by the pathologist, to follow-up with fluorescence *in situ* hybridization (FISH) and genetic analyses (e.g., oncotype), if indicated. All tissue samples are submitted for histopathological evaluation. The maximum pathological tumor size following VAE+CMSH is defined as the measure of the maximum size of the tumor in the slide of the greatest core sample compromised by the tumor. Following assessment, the VAE+CMSH pathology diagnosis (i.e., benign, B3 lesion, and DCIS or IC), the presence of DCIS with comedonecrosis, the biomarker status (e.g., ER/PR/HER2/Ki67), the morphological tumor type, and the nuclear and histological grades are all recorded.

### Surgical pathology

Gross surgical specimens are measured, weighed, and inked. All surgically excised tissue is submitted for histopathological evaluation, and slices are cut every 4 μm. Tests range from the usual HE analysis on slides, with or without immunohistochemistry and at the discretion of the case by the pathologist, to follow-up with FISH and genetic analyses (e.g., oncotype), if indicated. Following assessment, the margins status, the maximum pathological residual tumor size, the diagnosis (i.e., benign, B3 lesion, and DCIS or IC), the presence of DCIS with comedonecrosis, multifocality, the biomarker status (e.g., ER/PR/HER2/Ki67), the morphological tumor type, and the nuclear and histological grades are all recorded.

### Staging

All malignancies (DCIS/IC) are staged following the AJCC Cancer Staging Manual (26). Following the AJCC recommendation, the pathological tumor size (p*T*) based on gross measurement may be somewhat inaccurate. Microscopic assessment is preferred as it can distinguish fibrosis and noninvasive or invasive carcinoma. The microscopically determined p*T* is based on measurement of only the invasive component. For small invasive tumors that can be submitted in one section or paraffin block, microscopic measurement is the most accurate way to determine the p*T*. In some situations, systematic pathology evaluation allows microscopic reconstruction of the tumor; however, reconstruction measurements are correlated with the gross and imaging size before assignment of the p*T* (26). The AJCC emphasizes that, in patients who have undergone diagnostic vacuum-assisted core needle biopsy (VAB) sampling prior to surgical excision, measuring only the residual tumor may result in the underclassification of the T category and the understaging of the tumor, particularly with smaller tumors. In such cases, the original invasive cancer size is estimated and verified based on the best combination of the imaging, gross, and microscopic histological findings. Adding the maximum invasive cancer dimension on the VAB to the residual invasive tumor in the excision is not recommended as this method often overestimates the maximum tumor dimension. In general, the maximum dimension in either the VAB or the excisional biopsy is used for T categorization, unless the imaging dimensions suggest a larger invasive cancer (26). In the VAE BREAST 01 trial, the above AJCC recommendations for small tumors and tumors submitted to VAB prior to surgery are applied.

### Adjuvant treatment and follow-up

All patients receive adjuvant systemic therapy and radiotherapy according to the Brazilian Guideline for Breast Cancer Diagnosis and Treatment from the Brazilian Health Department (24).

### Outcomes

#### Primary outcome measures

The sensitivity, specificity, accuracy, PPV, NPV, FNR, and FPR of VAE+CMSH in predicting complete excision of breast cancers smaller than 1.5 cm in a one-step diagnostic–treatment approach will be calculated.

#### Secondary outcome measures

1. Evaluate the VAE+CMSH protocol defined complications and their management: bruises, skin lacerations, and unsuccessful procedures. Specifically for bruises, a scale was developed for quantification and classification (**Table 1**). The need for surgical drainage of VAE hematomas is also recorded.
2. Evaluate acceptance, cosmetic results, and satisfaction using The Breast-Q Questionnaire ICHOM Pre- and Postoperative Scales (27) in patients who have undergone VAE+CMSH and therapeutic breast surgery. Patients will be interviewed after VAE+CMSH and after therapeutic breast surgery and the results compared.
3. Evaluate the incidence of benign lesions, B3 lesions, and breast cancers (DCIS/IC) in ACR BI-RADS category 4–5 lesions ≤1.5 cm in mammography or ultrasound in the recent era.

**Table 1:** Bruising scale after vacuum-assisted excision (VAE).

Bruising scale	Findings	Physical examination
Grade 0	No bruises at all.	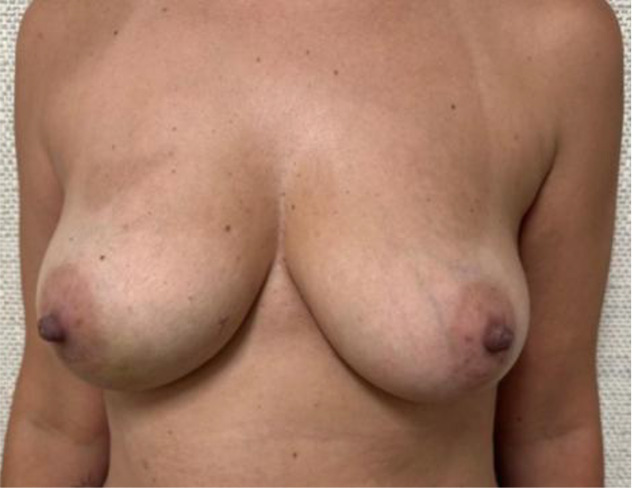
Grade 1	Mild bruises, including ordinary Tru-Cut core needle biopsy, on the left breast	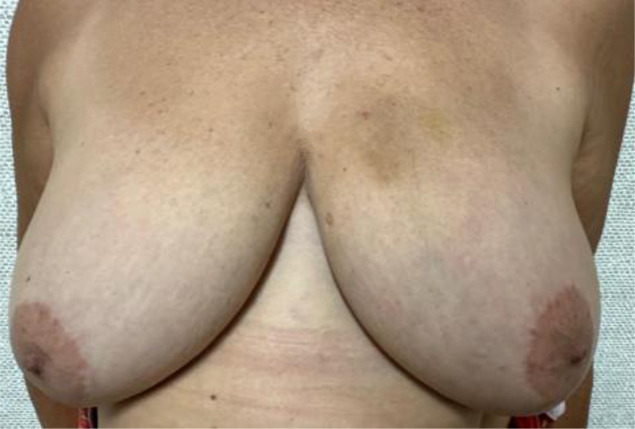
Grade 2	Moderate bruises, palpable nodular hematoma with localized skin bulging at the site of the procedure on the left breast	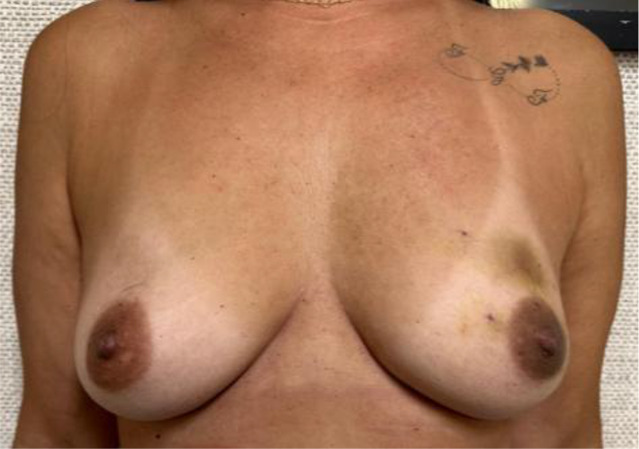
Grade 3	One quadrant extension bruises on the left breast	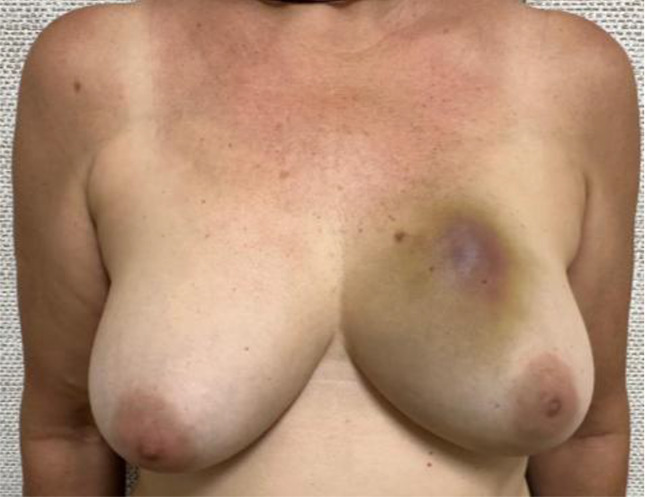
Grade 4	More than one quadrant bruise extension on the right breast	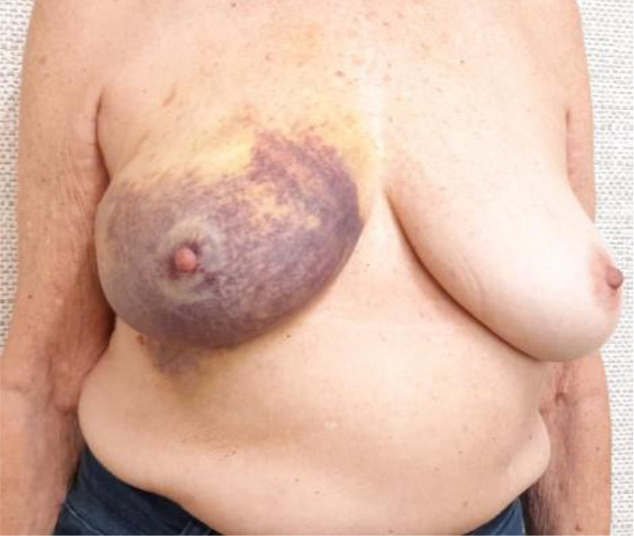

### Sample size calculation

The total sample size to be recruited is 353 patients based on a PPV_3_ of 35.9% (range = 20%–40%) for cancer in biopsies of ACR BI-RADS category 4–5 lesions (23), a margin of error of 5%, a 95% confidence interval, a FNR of the diagnostic test below 10%, and the availability of 100 positive cases. The 10% FNR refers to the minimum standard threshold at which all breast cancer de-escalation strategies have been proven effective (28).

### Healthy economic outcomes

If VAE+CMSH is proven to be an effective approach, the cost-effectiveness of upfront VAE+CMSH for the one-step diagnosis and excision of ≤1.5-cm ACR BI-RADS category 4–5 lesions *versus* the traditional CNB and subsequent surgery approach, when indicated, will be estimated based on the Brazilian Data on Breast Cancer (29), the Brazilian Methodological Guidelines for Economic Evaluation Studies of Health Technologies (30), and the recommendations from the National Commission for the Incorporation of Technologies into Unified Health System (CONITEC) (31), the Brazilian Public Health System (SUS).

### Mammographic and ultrasonographic image library

VAE BREAST-01 will generate a library of de-identified mammographic and sonographic breast images, with the aim being for future studies to identify potential image features that could determine cases where VAE+CMSH is associated with early breast cancer complete excision.

### Ethics and dissemination

Ethics approval was obtained from the Brazilian National Research Ethics Commission (CONEP). Participants will provide written informed consent, and researchers will follow institutional guidelines for data collection and management (Clinical Trial Registration: https://ensaiosclinicos.gov.br/; identifier: U1111-1301-4235). Data will be available at reasonable request to the chief investigator on completion of the trial and after publication of the results. The study results will be published in a peer-reviewed journal and presented at relevant specialty conferences. The findings will be shared with the relevant professional organizations to inform future guideline development.

## Discussion

Currently, there are several scenarios in which breast surgery can be omitted when lesions are treated using VAE: fibroadenomas and lesions of uncertain malignant potential are some examples (14–20). Active surveillance of DCIS with a low risk of progression to invasive cancer (32), percutaneous treatment of DCIS (33, 34), and small invasive breast tumors (21, 22, 35) are also under investigation.

The median breast cancer tumor size has decreased over the years due to mammographic screening. Breast cancers diagnosed in mammographic screening programs tend to be less aggressive luminal cancers, and some of them may represent overdiagnosis (3, 6, 7). Quality indicator goals for screening include a median tumor size of 14 mm, 77.3% node-negative cancers, 52.6% minimal cancers (<1 cm invasive cancers or *in situ*), and 74.8% stage 0 and 1 cancers (4, 23). The current guideline for axillary surgery recommends the omission of SNB in select patients who are postmenopausal and ≥50 years of age and in those with negative findings on preoperative axillary ultrasound for grade 1–2 small (≤2 cm), hormone receptor-positive, human epidermal growth factor receptor 2 (HER2)-negative breast cancers and who underwent breast-conserving therapy (11). A large portion of screen-detected breast cancer would be potentially excised using VAE, reducing the aggressiveness of surgical treatment and the impact of overtreatment.

Our group first study published evaluating the role of VAB (not VAE) in the excision of small malignant tumors reported 25% complete resection. However, in this series, CMSH was not obtained (21). Other studies have demonstrated that the use of VAB complete excision was possible in 18%–48.9% of cases (36–38), although none of these evaluated CMSH. Thus, the addition of the CMSH step could increase the chance and predict complete excision of a malignant tumor (35).

Free surgical margins are a determining factor in minimizing the local recurrence of breast cancer (39–42). However, the pursuit of wide margins must be balanced with the preservation of healthy tissue and the patient’s quality of life (43–46). Wider margins are unlikely to have additional benefits for the long-term local control of disease, and no ink on the tumor is sufficient for invasive disease, with a margin of 2 mm recommended for DCIS (39, 40). Analyzing this scenario, VAE+CMSH could be an approach for the complete excision of small breast cancers (DCIS/IC), minimizing the unnecessary excision of healthy tissue and reducing overtreatment.

It is a goal to reduce the healthcare interval of breast cancer. Delays in initiating breast cancer treatment are associated with significantly worse survival, particularly for cancer-specific mortality (47). Treatment interval (TI) is the time between the pathological diagnosis and the initiation of treatment (48). A recent meta-analysis has demonstrated that each additional 4-week delay in initiating treatment increases the risk of death by over 10%, underscoring the urgency of minimizing delays in diagnosis-to-treatment pathways (47). In Brazil, 21.5% of women with breast cancer take 31–60 days between diagnosis and treatment initiation, and 56.3% take more than 60 days (29). VAE+CMSH, as a one-step diagnosis and excision approach, could be useful in reducing the TI, particularly in countries where access to surgical treatment is difficult.

The incidence of B3 lesions varies between 3% and 21%, with higher rates in screening populations (49, 50). B3 lesions on CNB are currently mostly managed by second-line VAE (16–20). The estimated PPV_3_ of diagnostic imaging is 35.9% for cancer in biopsies of ACR BI-RADS category 4–5 lesions (23). There are no reliable data on the incidence of B3 lesions in VAEs of ≤15-mm ACR BI-RADS category 4–5 lesions. It is quite possible that more than 50% of these biopsies will present a B3 lesion diagnosis or small breast cancer. If upfront VAE+CMSH is successful in excising these lesions with free margins, this benefit could outweigh the adverse effects of, eventually, a more extensive procedure compared with ordinary Tru-Cut CNB for benign lesions.

There is great concern among surgeons whether VAE hematoma could lead to wider breast procedures or increase the mastectomy rate or compromised margins in breast cancer excisions after VAE-CMSH. In fact, the hematoma is confined to a cavity that contains the clip. The appropriate management of breast surgical excision after VAE-CMSH is excision of the cavity containing the clip. This excision can be performed at any time following VAE-CMSH, depending on the decision of the attending surgeon. Typically, even grade 3 or 4 hematomas are resolved within 30–60 days (51). The study will record the interval between VAE and surgery, the type of surgery performed (either breast-conserving surgery or mastectomy), the indication for each procedure, and the incidence of compromised surgical margins on breast surgery after VAE-CMSH.

There is the concern about completely excising an invasive triple-negative (TNBC) or HER2-positive breast cancer, which should be submitted to neoadjuvant chemotherapy (NAC) instead of upfront surgery. The majority of screen-detected small breast cancers are hormone receptor-positive cancers with good prognosis. TNBC or HER2-positive breast cancer is not generally diagnosed by mammographic screening or is ≤15 mm (6, 52). Early breast cancer guidelines recommend NAC for HER2-positive stage II or III breast cancer (>20 mm or N1) (8, 53).

HER2-positive breast cancers ≤15 mm associated with metastatic axillary nodes are candidates for NAC (8, 53). After VAE-CMSH diagnosing IC, the patient is staged, including axillary ultrasound evaluation and immunohistochemistry. In the case of pT1N1 HER2-positive IC, the patient is excluded and indicated for NAC. After NAC, the clipped breast tumor bed and axillary nodes are evaluated for residual disease according to standard of care (8, 53).

Early breast cancer guidelines recommend upfront surgery for ≤10-mm TNBC. In general, for T1c TNBC, the usual recommendation is NAC (8, 53). Nevertheless, no prospective double-blind randomized clinical trial has evaluated upfront surgery followed by NAC *versus* NAC followed by surgery for T1cN0 TNBC. Retrospective data showed that T1N0 TNBC submitted to upfront surgery followed by adjuvant chemotherapy did not have inferior outcomes compared with those who received NAC followed by surgery. However, patients with T1c tumors who achieved complete pathologic response to NAC had the best outcomes, suggesting that NAC provides important prognostic information that can guide adjuvant treatments (54). However, based on the results of CREATE-X, adjuvant capecitabine leads to improvements in overall survival (OS) and invasive disease-free survival (DFS) in patients with early TNBC and evidence of residual disease following NAC, with the population of T1 patients being poorly represented (14.7%) (55). Specifically, in relation to T1c (11–15 mm) TNBC, the data are scarcer. On the other hand, there is good evidence that delaying the initiation of adjuvant chemotherapy is critical for TNBC, particularly for stage IA (T1N0) (56). The diagnosis and treatment of ≤15-mm TNBC with a one-step procedure could reduce the time to adjuvant chemotherapy and improve prognosis.

## Conclusion

This trial design will provide an opportunity to standardize VAE associated with the CMSH procedure, as well as will evaluate its efficacy in achieving complete excision with clear margins in small breast cancers and other breast lesions ≤1.5 cm. The results of this trial will be crucial for advancing the de-escalation of breast cancer treatment.
